# Use of virtual reality compared to the role-playing methodology in basic life support training: a two-arm pilot community-based randomised trial

**DOI:** 10.1186/s12909-023-04029-2

**Published:** 2023-01-23

**Authors:** M Figols Pedrosa, A Barra Perez, J Vidal-Alaball, Q Miro-Catalina, A Forcada Arcarons

**Affiliations:** 1 Gerència Territorial de La Catalunya Central, Institut Català de la Salut, Sant Fruitós de Bages, Spain; 2 Servei d’Atenció Primària Bages-Berguedà-Moianès, Institut Català de la Salut, Manresa, Spain; 3 Unitat de Suport a la Recerca de la Catalunya Central, Fundació Institut Universitari per a la Recerca a l’Atenció Primària de Salut Jordi Gol i Gurina (IDIAPJGol), Sant Fruitós del Bages, Spain; 4 Health Promotion in Rural Areas Research Group, Gerencia Territorial de la Catalunya Central, Institut Català de la Salut, Sant Fruitós de Bages, Spain; 5 Faculty of Medicine, University of Vic-Central University of Catalonia, Vic, Spain

**Keywords:** Primary Care, Virtual Reality, Resuscitation, Learning, Teaching

## Abstract

**Introduction:**

Virtual reality (VR) is a technology that allows us to replace our real environment with one created with digital media. This technology is increasingly used in the training of healthcare professionals, and previous studies show that the involvement and motivation of students who participate in activities that use VR increases compared to those who undergo training with the traditional methodology. The main aim of the study is to evaluate the learning curve of the students using a VR environment, to evaluate the satisfaction with the training activity and the cost, and to compare them with training that uses role-playing methodology.

**Methodology:**

Two-arm community-based randomised trial. The control arm will base the training on the usual role-playing methodology. The second arm or intervention arm will base the Basic Life Support (BLS) training on a VR programme.

**Results:**

Statistically significant differences are observed in the percentage of correct answers in favour of the group that used VR as a learning methodology in the test taken at the end of the course. These differences disappear when comparing the results of the test performed at six months. The satisfaction rating of the role-playing training activity has a score of 9.37 out of a total of 10 and satisfaction with the VR methodology has a score of 9.72. The cost analysis shows that the cost of training a student by role-playing is 32.5 euros and, if trained by VR, it is 41.6 euros.

**Conclusions:**

VR is a tool that allows the consolidation of a greater amount of knowledge in the short term and can be used for situations such as pandemics, where traditional formats are not available. In relation to student satisfaction with the training activity, the rating in both groups is very high and the differences are minimal. The results will be directly applicable to the decision making of BLS training in Central Catalonia in relation to the scheduling of training activities that use the VR methodology in an uncertain environment.

## Background

The current economic context, characterised by globalisation, competitiveness and the progress of information and communication using new technologies, suggests that one of the critical elements for the development of organisations is knowledge and its management [1, 2]. This management is even more relevant for health services and health professionals due to the continuous development of knowledge in the health sciences. In this context, the incorporation of new training methodologies in companies becomes an interesting area of study [3, 4].

Numerous studies indicate that VR technology is a powerful tool for teaching, mainly because of its ability to provide immersive, multi-sensory and realistic teaching environments, among other features [5–7]. To achieve this immersion, we need to make use of technology and devices such as VR headsets or glasses and physical elements with which to interact with the virtual environment [6, 8]. Using these elements, we can transport ourselves to a world generated with the use of technologies.

There are studies that demonstrate the effectiveness of these programs in learning by studying the number of correct answers given by students before and after the intervention [9, 10]. However, they are circumscribed to the university environment, carried out with nursing, pharmacy or dentistry students, and do not refer to working professionals in the healthcare environment. These studies also do not assess the learning curve over a prolonged period.

The use of VR technology in the field of training is based on the theory that knowledge is retained much better when it is experienced directly than when it is simply observed or heard. The basis of this theory is the concept of first-person knowledge [5] according to which an individual acquires most knowledge during his or her lifetime through natural, direct, subjective and non-reflective experiences. Experiences of this type are usually characterised by the absence of deliberate reflection because the action arises directly from our perception of the world. Moreover, this learning happens implicitly, because we are not aware that we are learning something. In summary, VR allows the creation of first-person experiences, originally accessible through direct experience with the real world [11] and represents a very large qualitative leap in the learning of areas in which knowledge is difficult to visualise if it is not carried out in a simulation environment.

The use of VR in the field of education is gaining prominence [12] because it is an effective modality for training and assessment [13], which generates advantages for students [14], including understanding of the content presented [15] improved creativity, higher academic performance [6, 16], and increased student engagement in classrooms and ease of sustaining attention [17].

However, there are studies that have not observed a significant change in acquired knowledge between training with VR versus traditional training [17].

The application of VR in training is currently occurring mainly in the healthcare field [18, 19]. In this aspect, its usefulness is emphasised when making diagnostic or therapeutic decisions in health interventions, in the broadest sense of the term [20]. One of the fields of application of VR in the world of education is the use in the training of assistance techniques such as Basic Life Support (BLS) [21]. The Catalan Resuscitation Council (CCR) establishes that BLS training should be carried out with a 5-h programme. Every two years, professionals who have completed the initial certification course must undergo a recertification process. This information is available on the CCR website, which is part of the European Resuscitation Council (https://ccr.cat/).

The BLS training programme for Catalan Health Institute (ICS) professionals in Central Catalonia is endorsed by the CCR and consists of a five-hour certified training programme and a short recertification programme. The main objectives of this training are the knowledge of the action algorithm of a professional of the Catalan Health Institute in a cardiopulmonary arrest and training in resuscitation, evaluating as indicators of achievement a correct depth and rhythm of cardiac compressions performed by the student. Within this framework, Central Catalonia has initiated a training project for BLS certification using VR equipment.

The Catalan Health Institute (ICS) in Central Catalonia has a total of 33 Primary Care Teams (PCT) with a high rate of dispersion among the regions of Bages, Moianès, Berguedà, Osona and Anoia. This dispersion means that classroom training requires the students and/or teachers to travel.

The main aim of the project is to evaluate the learning curve of the students using a training environment with role-playing methodology and a VR environment, evaluating the consolidated learning contents after each training activity. The evaluation will be carried out through the questionnaire validated by the CCR and by the ERC, which will be given to the students immediately before the beginning of the classroom part of the training activity, immediately after six months of the course. The secondary aims are to evaluate the satisfaction of trainees who undergo training using VR versus a traditional methodology and to analyse the cost of training using VR and compare the cost using a conventional methodology.

## Methods

### Study design

Two-arm pilot community-based randomised control trial for the evaluation of a training intervention.

### Sample and sampling method

Professionals from the primary healthcare teams working in the healthcare facilities of the Catalan Health Institute of the Central Catalonia Territorial Management enrolled in the BLS certification courses that were scheduled in the different health care centres. The University Institute for Research in Primary Health Care Jordi Gol i Gurina (Barcelona, Spain) ethics committee approved the trial study protocol (approval code: 20/195-P).

The study is based on two arms: a control arm, which followed training in the usual role-playing methodology, and a second intervention arm, which followed BLS training with a VR programme.

In the total of 20 Basic Life Support certification training activities in the different territories organised by the training unit during the study period (March-December 2021), the intervention and control groups were randomly distributed as follows:

- 8 intervention groups in the regions of Bages, Berguedà and Moianès.
- 8 control groups in the regions of Bages, Berguedà and Moianès.
- 2 intervention groups in the region of Anoia.
- 2 control groups in the region of Anoia.

A total of 60 students were expected to attend each the intervention and control groups (6 students per group), with a maximum loss of 5% of enrolled students due to unforeseen circumstances, usually of a healthcare nature.

Actions carried out within the control arm. The subjects who underwent BLS training according to the usual methodology received a 15-min MS PowerPoint presentation prepared by the CCR, followed by a role-playing simulation using a sensorized torso and with the trainees performing the functions of simulation training. The total duration of this part was 5 h.

Actions carried out within the intervention arm. The subjects received BLS training based on a self-training strategy that consisted of using technological and computerised VR material. In this first phase, the training was carried out in group format. Once the training was completed, the students, individually, carried out a practical test using the virtual reality goggles. This second phase had a duration of 30 min per simulation scenario, using a minimum of two scenarios. The training was conducted at the workplace (health centres) and each student was assisted by a qualified instructor. The duration of the training was also of 5 h. The VR equipment consisted of a high-performance personal computer, a professional simulation torso with sensors, HTC Vive Cosmos® VR goggles and a carrying case. The software used was the one available in the virtual reality platform LUDUS (https://www.ludusglobal.com) that had a contract with our company during the year 2021. This platform provides of a repository of 5 virtual scenarios in simulation, located in a company, on the street, in a health centre, in a commercial space and in a public site with adverse weather factors. Each student could choose two scenarios to be used in the simulation. This platform has proven experience in the field of virtual reality training content edition.

### Study variables

The main outcome in the two arms of the training intervention is the learning curve of the students. This learning will be assessed by means of the Catalan Resuscitation Council's knowledge questionnaire and will be administered on three occasions: at time zero – before starting the training – to assess participants' baseline knowledge, immediately after completion of the training and six months after finishing the course. For this third evaluation, a reminder will be sent to their corporate email with a link to the intranet page of the teaching unit where they can answer the questionnaire.

The study also aims to assess aspects of satisfaction with the training activity. For this reason, students will be asked to answer a questionnaire on the training received (Annex 1).

Economic aspects will also be evaluated. The researchers will record the time that each student in each arm dedicated to the training, the time dedicated by the teachers and their remuneration and, if applicable, the rental of the equipment used for the training. Once this record has been made, the costs will be compared between the two groups.

Different sociodemographic, academic and occupational variables were recorded for each of the participants in each of the two arms of the study in order to stratify the learning outcomes by their different values. The variables collected were: sex, age, professional profile (clinical assistant, social worker, citizen care or other), job position (primary care centre certified for teaching or not certified) and time (in years) spent working in healthcare.

### Statistical analysis

For descriptive analysis, we expressed continuous variables as median (1r-3r quantiles) and we summarised categorical variables as absolute frequency (percentage). A bivariate analysis of the characteristics of the participating students was carried out to verify that their sociodemographic, academic and occupational data were homogeneously distributed between the two arms.

The bivariate analyses were carried out with the statistical tests that corresponded to the nature of the variables analysed. For dichotomous or polytomous qualitative variables were used Chi Squared test, and for quantitative variables that were distributed or not according to the normal distribution were used Mann–Whitney or Independent T-Test. The normality of the main outcome variable (knowledge scores) was checked and the evolution of its curve (time 0, time 1, time 6 months) was analysed.

A paired comparison was performed for the results of the baseline knowledge test and the score obtained at the end of the training and between this and the score obtained in the questionnaire at six months. The results obtained in each of the test arms were compared. For independent comparisons were used Mann Whitney test, and for paired comparisions were used Wilcoxon test.

The data were analysed with R software, version 4.1.2 for statistical analysis in its most recent version, and the alternative hypotheses were accepted when the probability of an error was less than 5% (*p* > 0.05).

## Results

**Fig. 1:**
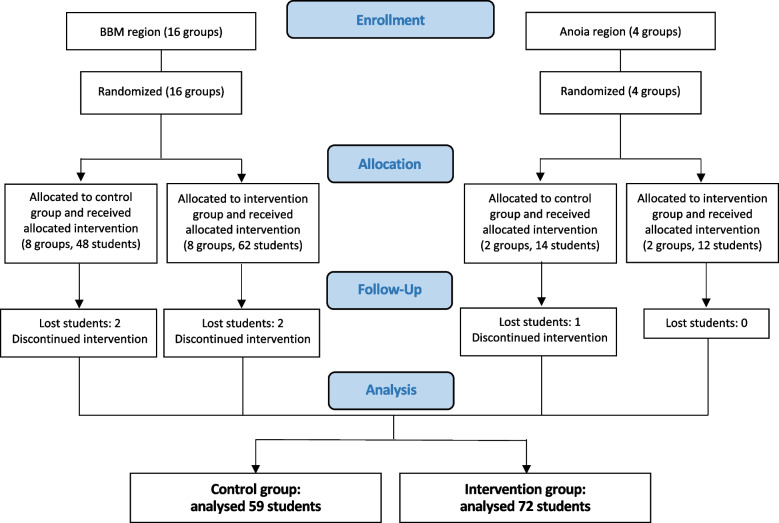
Flow diagram of BLS training activities and participants

Between March and July 2021, a total of 20 training activities were carried out, 10 activities for each of the intervention and control groups, with an average participation of 6.55 students per activity. In total, 62 subjects in the control group and 74 in the intervention group participated in the study. Of these, data from 59 subjects in the control group and 72 subjects in the intervention group were validated. There was a loss of 3 professionals in the control group and 2 professionals in the intervention group who had responded to the questionnaire at the start and end of the training but not to the six-month follow-up questionnaire (Fig. 1).

The variables of sex, age, profession, seniority of the professionals in the Catalan Health Institute and the certification of the centre where they work for the training of specialists in health sciences were collected (Table 1). It is shown that the distribution of the study subjects in each group is homogeneous.

**Table 1:** Description of the sample: absolute frequency and percentage

	**Intervention (*n* = 72)**	**Control (*n* = 59)**	***P*-value**
Sex			0.810^a^
Male	10 (16.9%)	10 (13.9%)	
Female	49 (83.1%)	62 (86.1%)	
Age (median and quartiles)	44.0 [33.0; 51.5]	38.0 [32.0; 48.0]	0.117^b^
Age			0.123^a^
Less than 30	9 (15.3%)	11 (15.3%)	
Between 30 and 40	13 (22.0%)	29 (40.3%)	
Between 40 and 50	17 (28.8%)	17 (23.6%)	
50 or more	20 (33.9%)	15 (20.8%)	
Years of service (median and SD)	3.0 [2.0; 8.5]	4.0 [2.0; 15.0]	0.251^b^
Years of service			0.724^a^
1 or 2 years	27 (45.8%)	28 (38.9%)	
3 or 4 years	9 (15.3%)	13 (18.1%)	
More than 5 years	23 (39.0%)	31 (43.1%)	
Certified PCT			0.875^a^
No	51 (86.4%)	64 (88.9%)	
Yes	8 (13.6%)	8 (11.1%)	
Profession			0.467^a^
Administrative staff	3 (5.08%)	8 (11.1%)	
Assistant	22 (37.3%)	17 (23.6%)	
Driver	0 (0.00%)	2 (2.78%)	
Diploma in Nursing	16 (27.1%)	15 (20.8%)	
Midwife	1 (1.69%)	0 (0.00%)	
GP	8 (13.6%)	12 (16.7%)	
Dentist	1 (1.69%)	2 (2.78%)	
Psychologist	0 (0.00%)	1 (1.39%)	
Patient Care Technician	6 (10.2%)	8 (11.1%)	
Technician	0 (0.00%)	1 (1.39%)	
TEGS	0 (0.00%)	3 (4.17%)	
Social Worker	2 (3.39%)	3 (4.17%)	

^a^Chi-Square test comparison

^b^Mann-Whitney test comparison

**Fig. 2:**
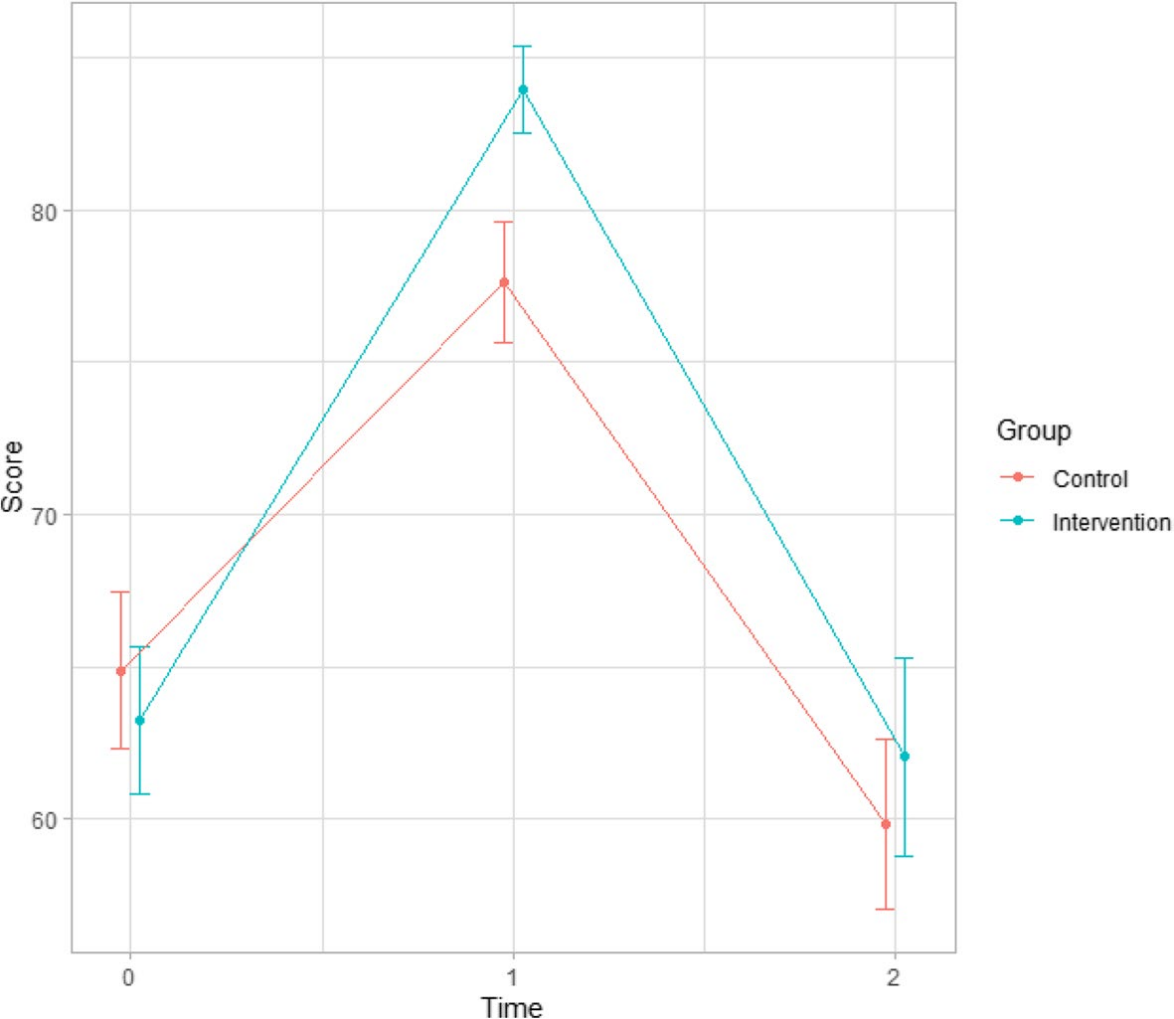
Scores evolution comparing control and intervention groups (mean and CI)

We also compared the results of the knowledge tests taken by the students prior to the start of the course, just after the end of the course and after six months. In this case, statistically significant differences are observed in the percentage of correct answers in favour of the group that used VR as a learning methodology in the test taken at the end of the course. These differences disappear when the results of the test performed at six months are compared (Table 2). The results of the pre-test show a percentage of correct answers in the control group of 64.9% compared to 63.3% in the intervention group (*p* = 0.618). In the test answered immediately after training, the percentage of correct answers in the control group was 76.4% and in the intervention group 84.0% (*p* = 0.036). In the tests performed at six months, the control group had a success rate of 59.8% and the intervention group 62.0% (*p* = 0.371). In relation to the curve of scores in Table 2 and Fig. 2, it can be seen that the greatest difference in scores between the two groups occurs in the test immediately following training. Results stratified by professional groups were not obtained.

**Table 2:** Score comparisons

	**Pre (int = 72, control = 59)**	**Post 1 (int = 72, control = 59)**	**Post 2 (int = 40, control = 28)**	**Comparison Pre vs Post 1**	**Comparison Pre vs Post 2**
Intervention	63.3 (20.6)	84.0 (12.2)	62.0 (20.5)	< 0.001^b^	0.191^b^
Control	64.9 (19.8)	77.6 (15.4)	59.8 (14.7)	< 0.001^b^	0.232^b^
Comparison	0.618^a^	**0.036**^**a**^	0.371^a^	-	-

^a^Mann-Whitney test comparison

^b^Wilcoxon test comparison

### Student satisfaction

In relation to the level of satisfaction of the students in each group, the intervention group has a score of 9.67 points out of a total of 10 points, and the control group an overall score of 9.39 points.

### Cost analysis

An analysis of the costs of each of the training methodologies has been carried out, extrapolating the data that the training carried out with the role-playing model and with the VR model entail for the organisation.

The role-playing training requires professionals to travel to a reference training centre. An average distance of 50 kms per professional has been calculated based on the average distance of the primary care teams in the training classroom. The cost per kilometre established by the public administration has been applied (reference: https://incasol.gencat.cat/web/.content/home/decret_indemnitzacions.pdf).

To this amount we added the cost of the teacher, according to the salary scale of the ICS professionals, and the reimbursement for the equipment used for training.

In VR training, the cost of equipment reimbursement and the cost of the software license have been accounted for because this method does not require the student to travel (Table 3).

**Table 3:** Cost comparisons

Costs per student	Control group	Intervention group
Equipment reimbursement	2.5 euros	13.1 euros
Software license		28.5 euros
Student travel	15 euros	
Teacher	15 euros	
**TOTAL**	**32.5 euros**	**41.6 euros**

The cost of the teacher is added only in the case of the control group training (15 euros per student), because in the intervention group the course is in self-training mode. The control group also has an expense of 15 euros per student for travel to and from the work station in the training room. This cost is due to the fact that primary care professionals in Central Catalonia are highly dispersed throughout the territory. Finally, a cost of 2.5 euros is added for reimbursement for the equipment used in the training of the control group. For the intervention group, the cost of equipment reimbursement (13.1 euros) and the software license (28.5 euros) was added.

## Discussion

The results of the study show that it is necessary for scientific societies to reflect on the recommendations for recertification of the knowledge acquired in the Basic Life Support course because the current recommendation is to do it every two years. This study can make us rethink this frequency and bring it closer to the training programme currently in force at the Catalan Health Institute, which recommends doing it annually.

No statistically significant differences were observed between content learning outcomes using a role-play methodology or using a VR methodology in training at that time of evaluation (*p* = 0.371).

A statistically significant difference (*p* = 0.036) in favour of VR was observed in the results of the knowledge test taken at the end of the course. This difference demonstrates that training using new teaching methodologies, in this case VR, allows for a better consolidation of knowledge in the short term among the students who participate. Further studies using other methodologies such as gamification are needed to validate other training models that provide greater consolidation of learning among students.

The improvement in the satisfaction rating observed between the two training methodologies, despite not having statistical evidence, does reflect what the rest of the studies and publications indicate in reference to an improvement in student satisfaction when using methodologies that allow them to interact and simulate real scenarios.

The costs of using the VR methodology are 9.1 euros higher per student than those of traditional training. This difference lies in the cost of licensing that product. If this training were to become widespread, it would lower the cost of the platforms and the gap would be expected to disappear. Other costs such as environmental costs have not been taken into consideration in this study. In this case, VR training can be expected to reduce costs for the environment by reducing student travel to classrooms where face-to-face training takes place.

VR allows the training of health service professionals, but it can have a more relevant importance during a period of restrictions such as the COVID-19 pandemic because each student can perform the practical part without the need to interact with other students [8, 22]. VR is a tool that allows self-training, but it can also be used in group training environments, so its potential as a teaching methodology is very important and it is advisable to extend its use, as well as that of other methodologies that can incorporate new technologies in the training process.

The study has some limitations. The availability of the equipment used to conduct the VR sessions may influence the scalability of the study. The study evaluated learning differences between the different professional groups working in the health centres but has not been able to find statistically significant results probably because the reduced number of subjects in each group. This study would have to be extended to obtain results in this sense. This could provide useful information on which professional groups this training could have the greatest impact and thus make the program more efficient.

## Conclusions

VR is a tool validated for the training of students in the healthcare environment because it allows the consolidation of a greater amount of knowledge in the short term and can be used for situations such as pandemics, where traditional formats are not available.

Student satisfaction when using participatory methodologies in training such as VR or role-playing is very high. It is necessary to extend the use of these methodologies in all training activities.

The cost difference between the two methodologies should disappear if organisations prioritise the use of VR as a teaching methodology due to the lower cost of content licenses.

## Data Availability

The datasets generated and/or analyzed during the current study are not publicly available as the contain information that could compromise the privacy of research participants but are available from the corresponding author on reasonable request.

## Abbreviations

VR: Virtual reality
BLS: Basic life support
CCR: Catalan resuscitation council
ICS: Catalan Health Institute
PCT: Primary Care Teams

## References

[1] Argyris C, Schön DA (1996). Organizational learning II: theory, method and practice.

[2] Davenport T, Prusak L (1998). Working Knowledge: How Organizations Manage What They Know.

[3] Kyndt E, Vermeire E, Cabus S (2016). Informal workplace learning among nurses: Organisational learning conditions and personal characteristics that predict learning outcomes. J Work Learn.

[4] Riera Claret C. Aprenentatge entre iguals i aprenentatge informal a l’organització. Plantejament d’un nou itinerari d’investigació per a l’Aprenentatge Organitzatiu [Internet]. TDX (Tesis Doctorals en Xarxa). Universitat de Vic - Universitat Central de Catalunya; 2013 [cited 2022 Jun 15]. https://www.tdx.cat/handle/10803/125862#page=1

[5] De Antonio Jiménez A, Villalobos Abarca M, Luna Ramírez E 2000 Cuándo y Cómo usar la Realidad Virtual en la Enseñanza. Rev Enseñanza y Tecnol 26–36. Available from: https://siteal.iiep.unesco.org/bdnp/393/plan-estrategico-gobierno-2015- 2019-solo-pais%0A. . https://www.nbn-resolving.org/urn:nbn:de:0168-ssoar-73414%0A. http://www.revmedmilitar.sld.cu/index.php/mil/arti

[6] Cagiltay NE, Ozcelik E, Berker M, Menekse Dalveren GG. The Underlying Reasons of the Navigation Control Effect on Performance in a Virtual Reality Endoscopic Surgery Training Simulator. Int J Human–Computer Interact [Internet]. 2019 14;35(15):1396–403. Available from: 10.1080/10447318.2018.1533151

[7] Pottle J. Virtual reality and the transformation of medical education. Futur Healthc J [Internet]. 2019 6(3):181–5. Available from: https://pubmed.ncbi.nlm.nih.gov/31660522 10.7861/fhj.2019-0036 PMC6798020 31660522

[8] Sivananthan A, Gueroult A, Zijlstra G, Martin G, Baheerathan A, Pratt P (2022). Using Mixed Reality Headsets to Deliver Remote Bedside Teaching During the COVID-19 Pandemic: Feasibility Trial of HoloLens 2. JMIR Form Res..

[9] Blanco-Ávila D, Gómez-Leal J, Sáenz-Montoya X. Increment del coneixement en suport vital bàsic facilitat per un recurs educatiu digital. Infermeria universitària, vol. 17, núm 1, pag. 42–53, 2020. [Access 12 19 2022] Available in: https://redalyc.org/journal/3587/358771757005/html/

[10] Tobase L, Peres HHC, Tomazini EAS, Teodoro SV, Ramos MB, Polastri TF (2017). Basic life support: evaluation of learning using simulation and immediate feedback devices. Rev Latino-Am Enfermagem.

[11] Vera Ocete G, Ortega Carrillo JA, Burgos González MÁ. La realidad virtual y sus posibilidades didácticas. Etic@.net [Internet]. 2003;2:2–17. Available from: http://www.ugr.es/~sevimeco/revistaeticanet/index.htm

[12] Panerai S, Catania V, Rundo F, Ferri R (2018). Remote Home-Based Virtual Training of Functional Living Skills for Adolescents and Young Adults With Intellectual Disability: Feasibility and Preliminary Results. Front Psychol.

[13] Abi-Rafeh J, Zammit D, Mojtahed Jaberi M, Al-Halabi B, Thibaudeau S (2019). Nonbiological Microsurgery Simulators in Plastic Surgery Training: A Systematic Review. Plast Reconstr Surg.

[14] Klippel A, Zhao J, Jackson K Lou, La Femina P, Stubbs C, Wetzel R, et al. Transforming Earth Science Education Through Immersive Experiences: Delivering on a Long Held Promise. J Educ Comput Res [Internet]. 2019 Jun 25;57(7):1745–71. Available from: 10.1177/0735633119854025

[15] Hanson J, Andersen P, Dunn PK (2019). Effectiveness of three-dimensional visualisation on undergraduate nursing and midwifery students’ knowledge and achievement in pharmacology: A mixed methods study. Nurse Educ Today.

[16] Jacobsen MF, Konge L, Bach-Holm D, la Cour M, Holm L, Højgaard-Olsen K (2019). Correlation of virtual reality performance with real-life cataract surgery performance. J Cataract Refract Surg.

[17] Lorenzo-Alvarez R, Rudolphi-Solero T, Ruiz-Gomez MJ, Sendra-Portero F (2019). Medical Student Education for Abdominal Radiographs in a 3D Virtual Classroom Versus Traditional Classroom: A Randomized Controlled Trial. AJR Am J Roentgenol.

[18] Vrillon A, Gonzales-Marabal L, Ceccaldi PF, Plaisance P, Desrentes E, Paquet C, et al. Using virtual reality in lumbar puncture training improves students learning experience. BMC Med Educ [Internet]. 2022;22(1):1–8. Available from: 10.1186/s12909-022-03317-7 10.1186/s12909-022-03317-7 PMC8981937 35379253

[19] Clarke E. Virtual reality simulation—the future of orthopaedic training? A systematic review and narrative analysis. Adv Simul. 2021;6:2. 10.1186/s41077-020-00153-x. https://advancesinsimulation.biomedcentral.com/articles/10.1186/s41077-020-00153-x. 10.1186/s41077-020-00153-x PMC7807709 33441190

[20] Fundación Estatal para la Formación en el Empleo. Evaluación de la calidad de las acciones de formación para el empleo en la modalidad de teleformación [Internet]. Vol. 2. 2020. Available from: https://www.fundae.es/docs/default-source/publicaciones-y-evaluaciones/evaluaciones/publicación-evaluación-de-la-calidad-de-la-teleformación-docx.pdf

[21] Issleib M, Kromer A, Pinnschmidt HO, Süss-Havemann C, Kubitz JC (2021). Virtual reality as a teaching method for resuscitation training in undergraduate first year medical students: a randomized controlled trial. Scand J Trauma Resusc Emerg Med.

[22] Buyego P, Katwesigye E, Kebirungi G, Nsubuga M, Nakyejwe S, Cruz P, et al. Feasibility of Virtual Reality based Training for Optimising COVID-19 Case Handling in Uganda. Res Sq [Preprint]. 2021 4:rs.3.rs-882147. 10.1186/s12909-022-03294-x PMC9006530 35418070

